# Synergistic drug combinations designed to fully suppress SARS-CoV-2 in the lung of COVID-19 patients

**DOI:** 10.1371/journal.pone.0276751

**Published:** 2022-11-10

**Authors:** Davide De Forni, Barbara Poddesu, Giulia Cugia, James Chafouleas, Julianna Lisziewicz, Franco Lori

**Affiliations:** 1 ViroStatics s.r.l., Tramariglio, Italy; 2 Research Institute for Genetic and Human Therapy, Colorado Springs, CO, United States of America; Rama Dental College and Hospital and Research Centre, INDIA

## Abstract

Despite new antivirals are being approved against SARS-CoV-2 they suffer from significant constraints and are not indicated for hospitalized patients, who are left with few antiviral options. Repurposed drugs have previously shown controversial clinical results and it remains difficult to understand why certain trials delivered positive results and other trials failed. Our manuscript contributes to explaining the puzzle: this might have been caused by a suboptimal drug exposure and, consequently, an incomplete virus suppression, also because the drugs have mostly been used as add-on monotherapies. As with other viruses (e.g., HIV and HCV) identifying synergistic combinations among such drugs could overcome monotherapy-related limitations. In a cell culture model for SARS-CoV-2 infection the following stringent criteria were adopted to assess drug combinations: 1) identify robust, synergistic antiviral activity with no increase in cytotoxicity, 2) identify the lowest drug concentration inhibiting the virus by 100% (LIC_100_) and 3) understand whether the LIC_100_ could be reached in the lung at clinically indicated drug doses. Among several combinations tested, remdesivir with either azithromycin or ivermectin synergistically increased the antiviral activity with no increase in cytotoxicity, improving the therapeutic index and lowering the LIC_100_ of every one of the drugs to levels that are expected to be achievable and maintained in the lung for a therapeutically relevant period of time. These results are consistent with recent clinical observations showing that intensive care unit admission was significantly delayed by the combination of AZI and RDV, but not by RDV alone, and could have immediate implications for the treatment of hospitalized patients with COVID-19 as the proposed “drug cocktails” should have antiviral activity against present and future SARS-CoV-2 variants without significant overlapping toxicity, while minimizing the onset of drug resistance. Our results also provide a validated methodology to help sort out which combination of drugs are most likely to be efficacious *in vivo*, based on their *in vitro* activity, potential synergy and PK profiles.

## Introduction

COVID-19 is still plaguing continents and crowding hospitals. Despite new antivirals such as molnupiravir and PF07321332-ritonavir have been recently approved, they suffer from significant shortcomings: 1) they carry a high pill burden (6 to 8 pills daily); 2) molnupiravir could induce genetic mutations and its efficacy is limited [1] 3) the threat of resistance is particularly severe for both such ‘monotherapies’ that each target only one part of the virus [2]; 4) they have not been authorized for initiation of treatment in patients hospitalized due to COVID-19 [3] because the benefit of treatment has not been observed in people when treatment started after hospitalization [4]. In addition, managing drug-drug interactions by ritonavir in the context of multi-treated hospitalized patients would be problematic. As most monoclonal antibodies have lost activity against the omicron variant [5], hospitalized patients with COVID-19 face disease progression and mortality with a scarce armamentarium of antivirals. The only small molecule available for them is remdesivir (RDV). RDV activity, however, has been inconsistent among clinical trials [6]. Other drugs have also been tested, mainly as add-on antiviral monotherapies, in SARS-CoV-2 infected patients, such as azithromycin (AZI) and ivermectin (IVM). Despite their benefits could not be ruled out, there was no evidence that they could provide a clear clinical advantage [7, 8]. The present study clarifies why the efficacy of either RDV or AZI or IVM has been inconsistent: each of these drugs needs relatively high concentrations to inhibit SARS-CoV-2 *in vitro* and their pharmacokinetic (PK) profile indicates that such therapeutically active concentration either cannot be reached or cannot be maintained in the fluids (e.g., plasma) and organs (e.g., lung) of human subjects, explaining their modest or null effect *in vivo* [9–13]. Our study also intends to offer a solution to overcome the problem: when the same drugs are used in combination, the concentration of each of these drugs needed to fully inhibit SARS-CoV-2 drops down to levels that are achievable in the lung for at least one day.

Antiviral drug monotherapies have already failed while drug combinations have already been successful against Human Immunodeficiency Virus, Hepatitis C Virus, and other viral epidemics by completely inhibiting viral replication, avoiding the onset of mutant viruses and drug resistance, preventing disease progression, and sometimes leading to virus eradication and cure [14–16]. Similarly, combining antivirals might lead to synergy and complete inhibition of SARS-CoV-2, the causative agent of the COVID-19 pandemic, especially when attacking the virus at multiple steps of its life cycle by broad-spectrum antivirals acting with different mechanisms of action such as RDV, AZI and IVM. In line with our hypothesis and proposed mechanism of action, a recent clinical study at the hospital of Parma, Italy, one of the first provinces hit by COVID-19 outside China in early 2020, showed that intensive care unit admission was significantly delayed by the combination of AZI and RDV, but not by RDV alone, in a stepwise multivariate logistic regression model [17].

Our suggested highly active broad-spectrum antiviral drug cocktails may have applications for the treatment of patients hospitalized with COVID-19. These patients have the highest need for effective antivirals for several reasons: 1) they carry a substantially higher viral load compared to non-hospitalized patients, 2) a higher viral load appears to be associated with more severe clinical outcomes and its reduction is most likely required for efficacy [18] and 3) they have limited antiviral options, presently only one antiviral (RDV) and one monoclonal antibody (sotrovimab). Our methodology also intends to offer a tool to predict which combinations of drugs will be likely efficacious in the clinics, limiting the number of drawbacks during clinical trials like has happened in the past when too many drugs that were promising *in vitro* failed to work *in vivo*.

## Materials and methods

### Drugs

Remdesivir (GS-5734, HY-104077) was purchased from MedChemExpress (Monmouth Junction, NJ). Azithromycin (CP-62993, S1835), ivermectin (MK-933, S1351), umifenovir (arbidol, S2120) and nitazoxanide (NSC 697855, S1627), hydroxychloroquine (NSC 4375, S4430), homoharringtonine (CGX-635, S59015), camostat (FOY-305, S2874), darunavir (S5250) and lopinavir (ABT-378, S1380) were purchased from SelleckChem (Houston, TX).

### Cell preparation

Vero E6 cells (Cercopithecus aethiops, kidney, ATCC CRL-1586) were seeded at a density of 10,000 cells/well into a 96-well plate in DMEM supplemented with 1% glutamine, 1% penicillin/streptomycin and 10% fetal bovine serum (complete medium, CM) at 37°C and 5% CO_2_. 24 hours after seeding fetal bovine serum concentration was decreased to 2% to avoid interference with the viral infection.

### Cytotoxicity of drugs

24 hours after seeding cells reached ~70% confluency and were treated with increasing concentrations of the drugs, alone or in combination, and cultured for additional 72 hours. At the end of the incubation period, the cytotoxic effect was measured through MTS assay (CellTiter 96® Aqueous One Solution Reagent, Promega) and by observation of cell monolayer integrity under light microscopy. Each compound was tested in duplicate wells to determine the HCC_0_, which is the highest concentration showing 0% cytotoxicity (i.e., 100% viability) compared to the untreated control.

### Antiviral activity of drugs

24 hours after seeding cells reached ~70% confluency and the supernatant in each well was replaced with 2% serum CM containing drugs at increasing concentrations, alone with each concentration tested in duplicate wells (typically 0.08 μM to 100 μM in 1:5 ratio dilutions), or in dual drug combination (creating a matrix of 49 different combinations of the two drugs using the same range of concentrations used for each of the drugs alone). 1 hour later cells were infected with SARS-CoV-2 at a multiplicity of infection (MOI) of 0.01 and cultured for 72 hours. Four strains were used: 1) the human 2019-nCoV strain 2019-nCoV/Italy-INMI1, isolated in Italy (ex-China) from a sample collected on January 29, 2020, kindly provided by the Istituto Lazzaro Spallanzani, Rome, Italy [19], 2) GZ69 (genomic data are available at EBI under study accession n. PRJEB38101), 3) Delta variant B.1.167.2 and 4) Omicron BA.1 variant, the last three viral strains kindly provided by the Department of Molecular and Translational Medicine, Section of Microbiology and Virology, University of Brescia Medical School, Brescia, Italy [20–23]. The 2019-nCoV/Italy-INMI1 strain was used for initial experiments and for drug combination studies, GZ69 was used as confirmatory strain as it was the main circulating variant in Italy at the time experiments were performed. Delta and Omicron strains were also used for confirmatory monotherapy experiments. At the end of the incubation period, viral replication was measured by cytoprotection assay through observation of cell monolayer integrity under light microscopy. Each compound was tested in duplicate wells to determine the LIC_100_, which is the lowest drug concentration that 100% inhibits virus replication. The Therapeutic Index (TI) was calculated as the ratio between HCC_0_ and LIC_100_.

As a validation of the cytoprotection assay readout, an ELISA test (SARS-CoV-2 Nucleocapsid Detection ELISA Kit, Sino Biological) was performed in monotherapy experiments on supernatants collected at the end of the culture period to measure produced virus through the quantification of the viral NP nucleoprotein (a measure of viral replication capacity).

### Drug combination experiments

**Fig 1:**
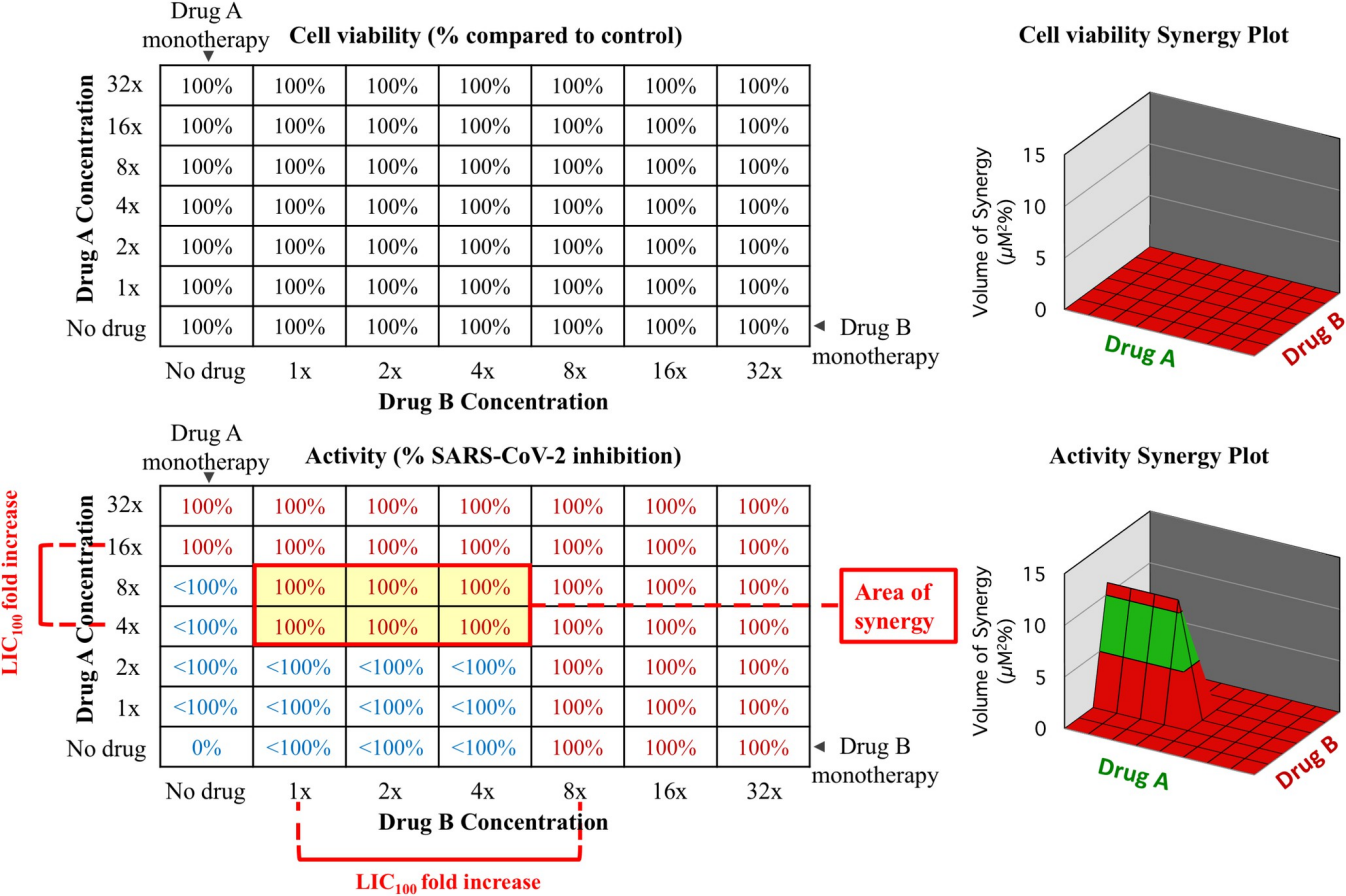
Conceptual design of evaluation matrix for antiviral activity and cytotoxicity of drug combinations *in vitro*. Cytotoxicity (upper left) is expressed as cell viability; Antiviral activity (lower left) is expressed as 100% or <100% inhibition of virus replication; LIC_100_ is the lowest drug concentration that completely inhibits virus replication; fold activity increase (fold changes in LIC_100_) and area of synergy are shown. The lower left well in each panel represents DMSO-containing medium with no drugs as control. 3D representations of the results are shown in the right part of the figure where synergy is only detected for activity, not for cytotoxicity.

For each drug combination the compounds were prepared separately by two-fold serial dilution and mixed in 96-well assay plates to create a two-dimensional matrix of diluted drugs generating up to 49 different combinations of two drugs plus a control without drugs Equal aliquots from the individual drug dilution plate were transferred to the cell-containing plates, cells were infected with the SARS-CoV-2 2019-nCoV/Italy-INMI1 strain at an MOI of 0.01 and incubated at 37°C and 5% CO_2_ for 72 hours. After incubation, cytotoxic effect and antiviral efficacy were evaluated as described previously. The raw data were analyzed using the MacSynergy II software that produces synergy plots representing the statistically significant synergy or antagonism at the level of 95%, 99% and 99.9% confidence, calculated with a Bonferroni adjustment. A 3D plot of the surface was generated using the intrinsic plotting software through Excel [24]. At least three independent experiments were conducted for each combination. **Fig 1** shows an example of Drug A and Drug B combination leading to synergy and lowering the LIC_100_ that resulting in a several-fold increase in the activity of both drugs. We first ensure that there is no increase in cytotoxicity when drugs are combined; every experiment with increased cytotoxicity in any of the wells analyzed is discarded. Then we compare the activity of two drugs (Drug A and Drug B) as monotherapy or in combination. The matrix allows for the determination of the area of synergy, the LIC_100_, which is the lowest drug concentration that completely inhibits virus replication and the LIC_100_ fold increase.

## Results and discussion

From over 100 publications describing at least one drug potentially able to inhibit SARS-CoV-2, we selected 26 drug candidates with well-documented antiviral activity. Each drug was tested as monotherapy for cytotoxicity and antiviral activity using the 2019-nCoV/Italy-INMI1 SARS-CoV-2 strain and 10 of them were further selected as their TI was above 1. These compounds were: hydroxychloroquine, umifenovir, nitazoxanide, azithromycin, ivermectin, homoharringtonine, camostat, remdesivir, darunavir and lopinavir. The acceptability criteria for these compounds were confirmed with the GZ69 SARS-CoV-2 strain. Similar results were obtained when remdesivir was tested against the delta and omicron variants (not shown). This was not unexpected, since immunological variants have emerged to escape the pressure induced by the immune system and not to become resistant to drugs.

We analyzed all potential double combinations of the 10 selected antivirals by using stringent criteria: 1) the Highest Concentration with 0% Cytotoxicity (HCC_0_), 2) the Lowest 100% Inhibitory Concentration (LIC_100_) and 3) the calculation of synergy by a rigorous and well-established model [24]. An accurate search of combinations with 0% cytotoxicity coupled with 100% viral inhibition *in vitro* are prerequisites to look for maximal safety and antiviral efficacy *in vivo* and are expected to bear clinical relevance.

**Fig 2:**
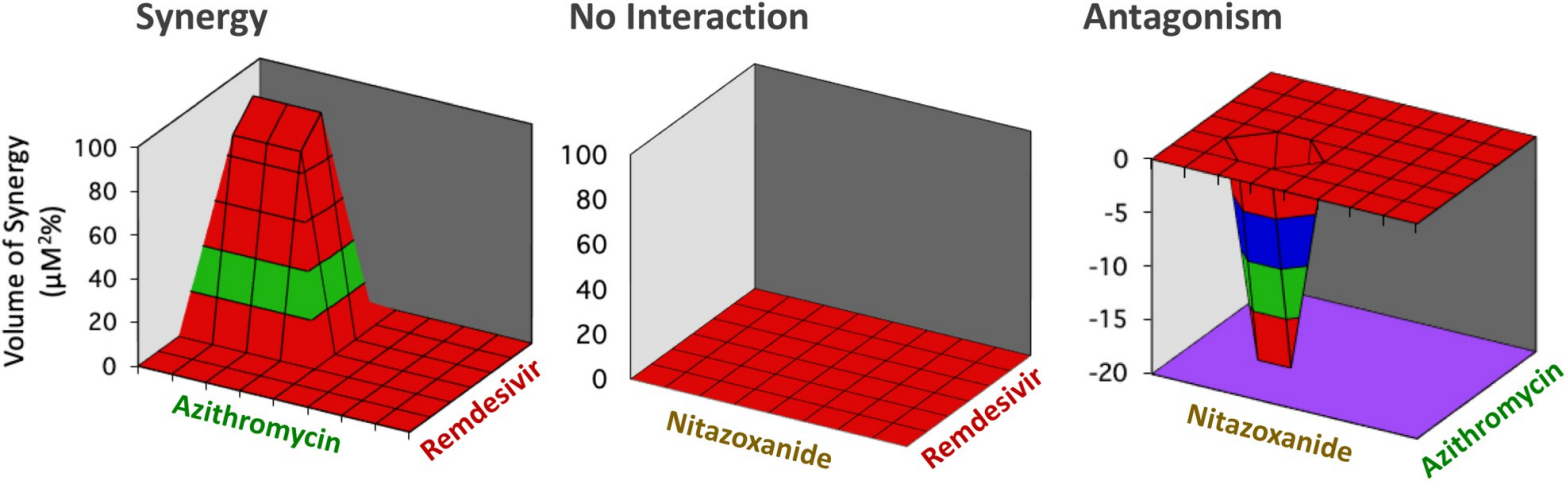
Antiviral activity of drug combinations. The model analyzes all the possible combinations of three drugs in a three-dimensional diagram (pyramid). Experimental outcomes: (left) synergistic effect, that is a pyramid up; (center) no interaction, that is no pyramid; (right) antagonistic effect, that is a pyramid down.

**Fig 2** illustrates the variety and unpredictability of the experimental outcomes. For example, by combining the same three drugs in the three possible double combinations we obtained three different results: remdesivir with azithromycin, remdesivir with nitazoxanide, and azithromycin with nitazoxanide led to synergy, no interaction or antagonism, respectively.

We then focused on the potential synergies of the remdesivir (RDV) based double combinations, as RDV was the only approved anti-SARS-CoV-2 drug among the selected antivirals. Both azithromycin (AZI) and ivermectin (IVM) showed synergistic inhibition of SARS-CoV-2 replication with RDV in the absence of any antagonism, as illustrated in **Table 1**.

**Table 1:** Synergistic antiviral activity of RDV-based cocktails.

	Synergy (μM^2^%)	Log Volume	Antagonism (μM^2^%)	Log Volume
**RDV + AZI**	578	54	0	0
**RDV + IVM**	950	49	0	0
**RDV + UMI**	734	69	0	0
**RDV + NTZ**	44	4	0	0
**RDV + HHT**	44	4	-355	-33
**RDV + CAM**	100	9	-122	-11
**RDV + DRV**	561	52	-22	-2

Scoring remdesivir (RDV) synergy (or antagonism) with different drugs: azithromycin (AZI), ivermectin (IVM), umifenovir (UMI), nitazoxanide (NTZ), homoharringtonine (HHT), camostat (CAM) and darunavir (DRV). According to the software manual, highly significant synergy (or antagonism) between two drugs is achieved when μM^2^%, is >100 (-100 for antagonism) and Log volume is >9 (-9 for antagonism).

**Fig 3:**
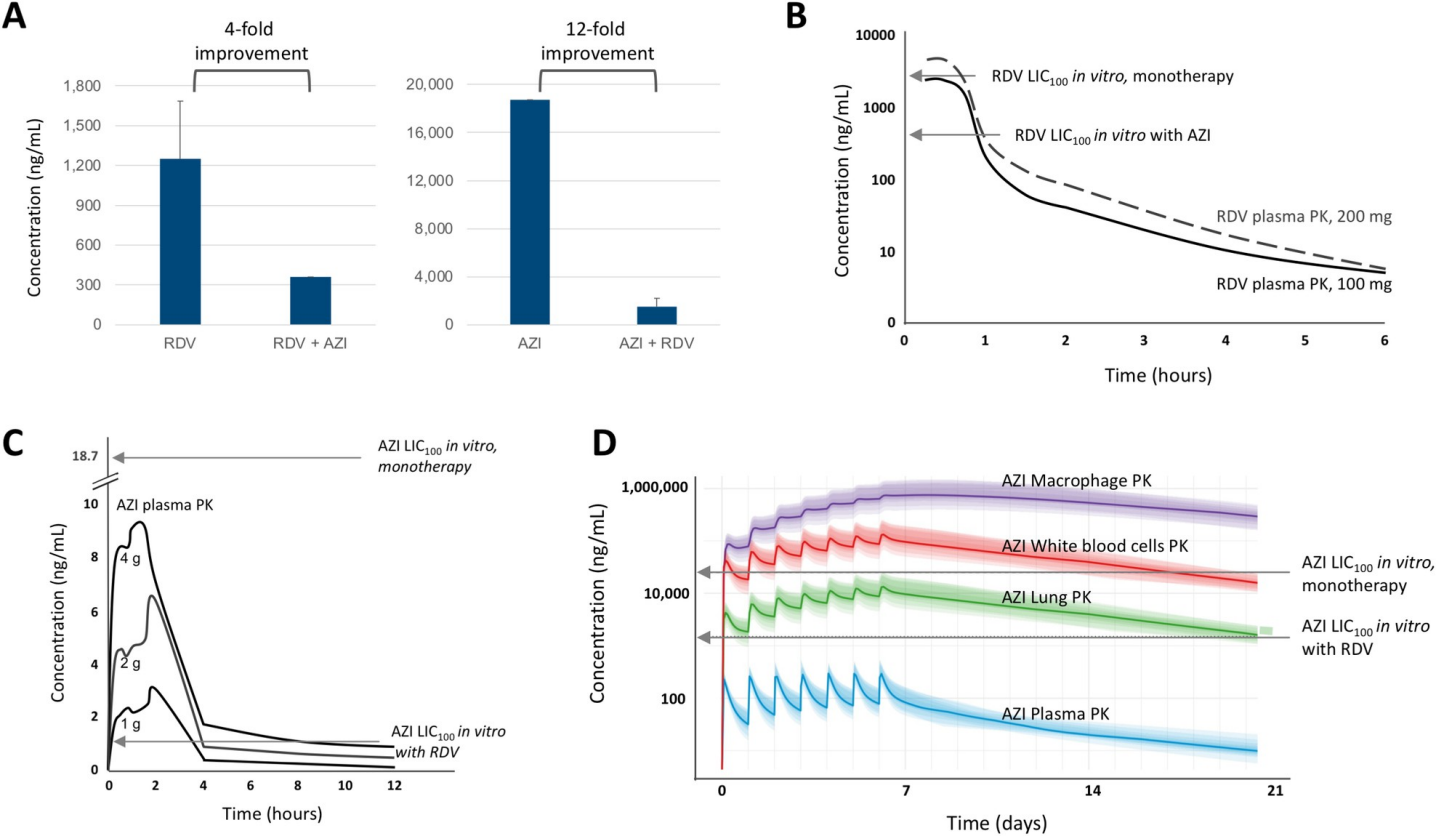
Analyses of RDV and AZI combination. (A) RDV and AZI lowest concentrations required for 100% inhibition of SARS-CoV-2 (LIC_100_) are lowered by an average of 4- and 12-fold, respectively, when the drugs are combined *in vitro*. (B) RDV antiviral activity *in vitro* vs. exposure *in vivo* in human plasma (PK data was adapted from [9]). RDV monotherapy *in vitro* has a LIC_100_ (upper arrow) that cannot be consistently reached in the plasma with standard I.V. dosages. Combining RDV with AZI reduces *in vitro* LIC_100_ (lower arrow) down to levels that can be consistently maintained in the plasma for at least 1 hour. (C) Antiviral activity of AZI *in vitro* vs. exposure *in vivo* in human plasma after I.V. administration (adapted from [12]). AZI monotherapy *in vitro* has a LIC_100_ (upper arrow) that cannot be reached in the plasma with standard (e.g., 1, 2, 4 grams) I.V. dosages. Combining AZI with RDV reduces the *in vitro* LIC_100_ (lower arrow) down to levels that can be maintained in the plasma for up to 8 hours. (D) Antiviral activity of AZI *in vitro* vs. exposure *in vivo* in human tissues after oral administration (calculated through Pfizer’s azithromycin PK Simulator [https://azpksim.pfizer.com]). AZI monotherapy *in vitro* has a LIC_100_ (upper arrow) that cannot be reached in the lung after per os administration of 500 mg AZI once daily for 7 days. Combining AZI with RDV reduces *in vitro* LIC_100_ (lower arrow) down to levels that can be maintained in the lung for up to 21 days.

The RDV and AZI synergistic combination led to a marked increase in potency, illustrated by a decrease in LIC_100_ (**Fig 3A**), meaning that a much lower concentration of each drug (4- and 12-fold for RDV and AZI, respectively) was needed to achieve the complete inhibition of virus replication when the drugs were combined compared to monotherapy.

Decreasing the LIC_100_ of a drug has important pharmacological implications, because it is equivalent to increasing the therapeutically active drug exposure in human subjects. For example, the LIC_100_ of RDV monotherapy *in vitro* is 1,250 ng/ml. Based on the pharmacokinetic profile of clinically indicated RDV doses [9] a 1,250 ng/ml concentration would be inconsistently reached and shortly maintained in the plasma (**Fig 3B**). The scenario changes when RDV is combined with AZI, as the resultant LIC_100_ of the RDV combination decreases to 360 ng/ml, a concentration of RDV that can be consistently maintained in the plasma for at least one hour.

For the treatment of all respiratory viruses, including SARS-CoV-2, the drug concentration reached in the lung is the most relevant one. Since RDV is a pro-drug, measuring its PK in the plasma is not sufficient. The active RDV metabolite is its nucleoside triphosphate form (Nuc-TP) that will accumulate intracellularly in the lung at 4–10 μM [13] following the administration of an RDV dose of 200 mg I.V. (**Table 2**, upper portion).

**Table 2:** Complete inhibition of SARS-CoV-2 replication in the lung is achievable by RDV + AZI and RDV + IVM, but not by RDV monotherapy.

From Sun *et al*. [13]	RDV (μM)	Nuc-TP (μM), estimated
Human lung, intracellular concentration	Not applicable	4–10
IC_50_ RDV	0.77	7.7
IC_90_ RDV	1.76	17.6
**From our experiments**		
LIC_100_ RDV	2.1	21
LIC_100_ RDV + AZI	0.6	6
LIC_100_ RDV + IVM	0.3	3

Central column: IC_50_, IC_90_ and LIC_100_ *in vitro* of RDV alone and in combination with either AZI or IVM; right column: estimated concentration of RDV active metabolite (Nuc-TP) in the human lung after a 200 mg dose exposure I.V. and estimated IC_50_, IC_90_ and LIC_100_ of Nuc-TP when its precursor RDV would be administered as monotherapy or in combination with either AZI or IVM.

It has been estimated that the IC_50_ and the IC_90_ for the Nuc-TP in the lung would be 7.7 μM and 17.6 μM, respectively (that is higher than the IC_50_ and IC_90_ for RDV *in vitro* by a factor of 10). These results explain the inconsistent efficacy of RDV in clinical practice, as hospitalized patients are exposed to a suboptimal Nuc-TP concentration, only able to inhibit 50% of SARS-CoV-2 replication. The LIC_100_ of RDV monotherapy *in vitro* was 2.1 μM, corresponding to an estimated concentration of Nuc-TP of 21 μM (**Table 2,** lower portion) that cannot be reached in the lung by RDV monotherapy [13]. In contrast, when tested in combination with AZI, the resultant RDV LIC_100_ decreased to 0.6 μM, corresponding to a Nuc-TP concentration of 6 μM, which is reachable intracellularly in the lung after administration of clinically indicated RDV doses. Since the Nuc-TP intracellular half-life is 14–24 hours, it is expected that SARS-CoV-2 would be exposed to therapeutically effective concentrations for the whole day with this combination therapy.

AZI antiviral activity also benefits from the combination with RDV to an extent that becomes relevant *in vivo*. Commonly used antibacterial doses of AZI have not shown anti-SARS-CoV-2 activity in human subjects [25] because the therapeutically active anti-SARS-CoV-2 concentration (LIC_100_) cannot be achieved in the plasma even by 1 to 4 gr AZI I.V. doses [12]. However, when AZI would be administered in combination with RDV, the resultant LIC_100_ of AZI would decrease to a concentration of 1,500 ng/mL that can be maintained for 4 to 8 hours in the plasma (**Fig 3C**).

When administered orally the AZI exposure in the plasma is lower than after I.V. administration (**Fig 3D**) and the AZI LIC_100_ values remain higher than the AZI concentrations, regardless of whether AZI is used as monotherapy or in combination with RDV. However, for the treatment of respiratory viruses what is most relevant is reaching an effective drug concentration in the lung, as complete inhibition of SARS-CoV-2 is expected if the drug concentration in the lung remains higher than the LIC_100_. While AZI monotherapy has a LIC_100_ that cannot be reached in the lungs after oral administration of commonly used AZI doses (e.g., 500 mg once daily for 7 days), when used in combination with RDV the resultant LIC_100_ of AZI drops to a concentration of 1,500 ng/mL that can be maintained in the lungs up to 21 days (**Fig 3D**).

**Fig 4:**
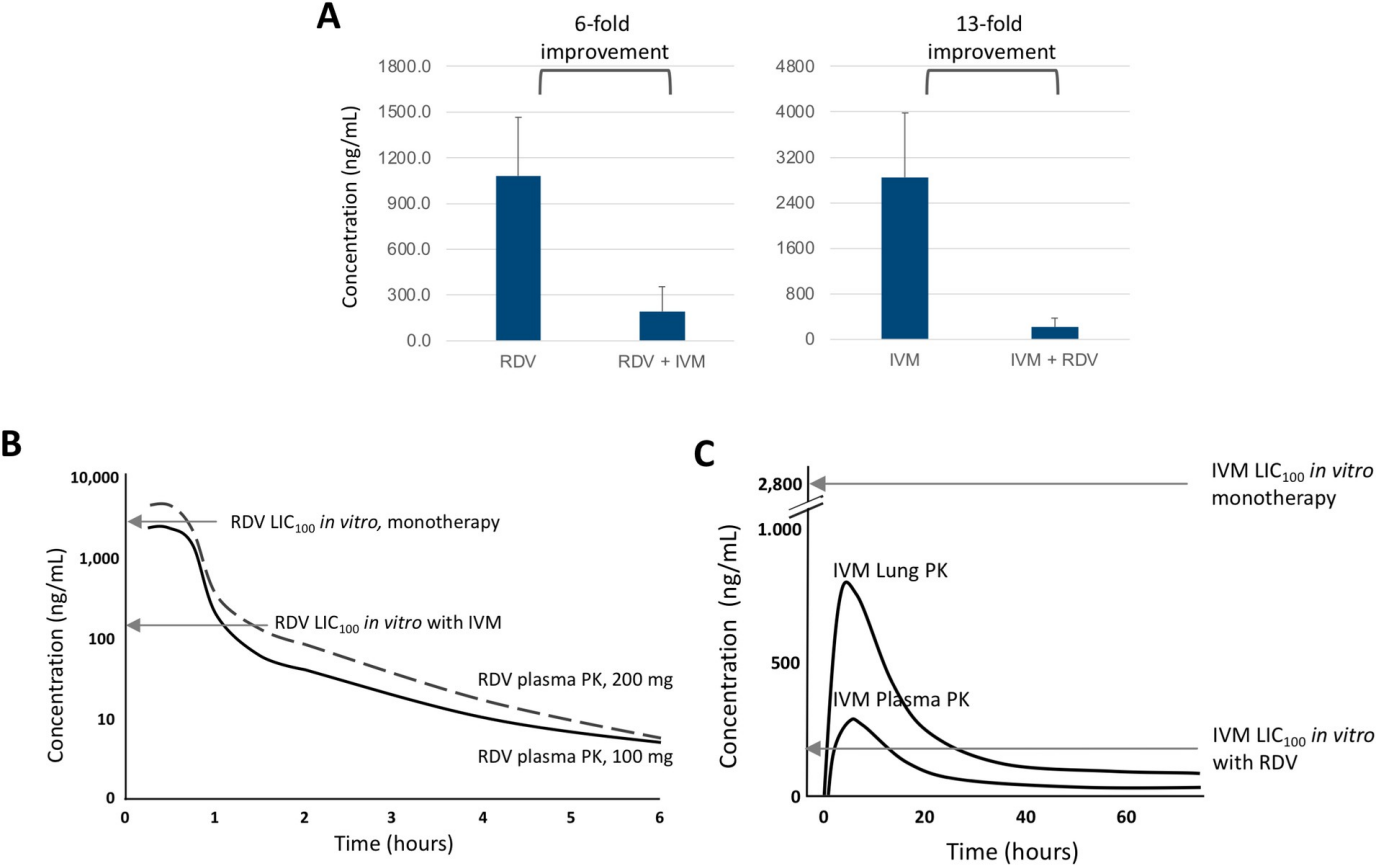
Analyses of RDV and IVM combination. (A) RDV and IVM lowest concentrations required for 100% inhibition of SARS-CoV-2 (LIC_100_) are lowered by an average of 6- and 13-fold, respectively, when the drugs are combined *in vitro*. (B) RDV antiviral activity *in vitro* vs. exposure *in vivo* in human plasma (PK data was adapted from [9]). RDV monotherapy *in vitro* has a LIC_100_ (upper arrow) that cannot be consistently reached in the plasma with standard I.V. dosages. Combining RDV with IVM reduces *in vitro* LIC_100_ (lower arrow) to levels that can be consistently maintained in the plasma for at least 1.5 hours. (C) Antiviral activity of IVM *in vitro* vs. exposure *in vivo* in the plasma and the lung (adapted from [11]). IVM monotherapy *in vitro* has a LIC_100_ (upper arrow) that can be reached neither in the plasma nor in the lung with standard oral dosages. Combining IVM with RDV reduces the *in vitro* LIC_100_ (lower arrow) to levels that can be maintained in the plasma for 16 hours and in the lung for 30 hours (blue dotted line). Both LIC_100_ values and plasma concentrations are expressed as ng/mL.

The RDV and IVM combination also results in a synergistic increase in anti-SARS-CoV-2 activity (**Table 1**) which leads to a marked decrease in LIC_100_, meaning that a much lower concentration of each drug (6- and 13-fold lower for RDV and IVM, respectively) was needed to achieve the complete inhibition of virus replication when the drugs were combined compared to monotherapy (**Fig 4A**).

**Fig 4B** illustrates the potential clinical implications of the *in vitro* results. Compared to monotherapy, in combination with IVM, the resultant LIC_100_ of RDV decreases from a concentration unlikely to be consistently achieved or maintained in the plasma after administration of commonly used RDV doses [9] to a concentration that can be consistently maintained in the plasma for one and half hour. RDV LIC_100_ *in vitro* as monotherapy was 2.1 μM, corresponding to an estimated concentration of its active triphosphate form (Nuc-TP) of 21 μM, not reachable in the lung [13]. In combination with IVM, the resultant RDV combination LIC_100_ decreased to 0.3 μM, corresponding to a Nuc-TP concentration of 3 μM, attainable intracellularly in the lung (**Table 2**) and expected to be maintained for 14–24 hrs. The combination of RDV and IVM also enhances the antiviral activity of IVM. When IVM is administered as monotherapy, the LIC_100_ is not reachable in the plasma and in the lung even with the highest 120 mg dose [11]. In combination with RDV, the resultant LIC_100_ of IVM decreases to 213 ng/mL, a concentration reachable in the plasma for 12 hours and in the lung for 24 hours (**Fig 4C**).

In summary, combining RDV with either AZI or IVM (all with suboptimal exposure in the plasma as monotherapies) would result in exposure to therapeutically effective antiviral doses of each drug in the plasma. When RDV, AZI, or IVM would be administered as monotherapy, only RDV would be partially active in the lung. However, when administered in combination, both drugs of the respective combinations would achieve their therapeutically effective antiviral concentrations in the lung with the resultant antiviral activity expected to last for a whole day.

Finally, combining RDV with either AZI or IVM increases each drug’s activity without increasing cytotoxicity and therefore substantially broadens the TI of each drug, from 14-fold (least good scenario) to 32-fold (best case scenario) (**Table 3**).

**Table 3:** Increased activity and broadened Therapeutic Index (TI) by combining RDV with either AZI or IVM. Upper Table: Comparison of the effect of drug combinations on antiviral activity and cytotoxicity. Lower Table: TI is calculated here as the ratio between the highest HCC_0_ and the lowest LIC_100_ as monotherapy and among all RDV-based combinations.

**Upper Table**												
				**RDV + AZI**					**RDV + IVM**			
**Activity Increase**				RDV			4X		RDV			6X
**(LIC**_**100**_ **fold decrease)**												
**Relative to Monotherapy**				AZI			12X		IVM			13X
**Cytotoxicity**				RDV			0%		RDV			0%
**(% Dead Cells vs Control)**												
**Relative to Monotherapy**				AZI			0%		IVM			0%
**Lower Table**												
**Drug**	**Cytotoxicity and activity range (μM)**					**Therapeutic Index (TI)**					**TI Fold Increase**	
	HCC_0_	LIC_100_ Monotherapy	LIC_100_ Combination		TI			TI		Combination/		
					Monotherapy			Combination		Monotherapy		
**RDV**	5.0	2.5	0.18		2			28		14		
**AZI**	50.0	25.0	2		2			25		13		
**IVM**	4.0	4.0	0.125		1			32		32		

Legends: HCC_0_ = Highest concentration with 0% cytotoxicity; LIC_100_ = Lowest concentration with 100% inhibition; TI = ratio between HCC_0_ and LIC_100_

Broadening the TI provides a larger window of opportunities and additional flexibility to the clinicians that could adjust drug dosages as needed with less concern for side effects. If a broader TI is achieved while increasing drug potency (as it was the case in our combinations) the drug dose could even be decreased while maintaining efficacy against SARS-CoV-2, for example in the case of the onset of reversible side effects, instead of withdrawing treatment.

**Fig 5:**
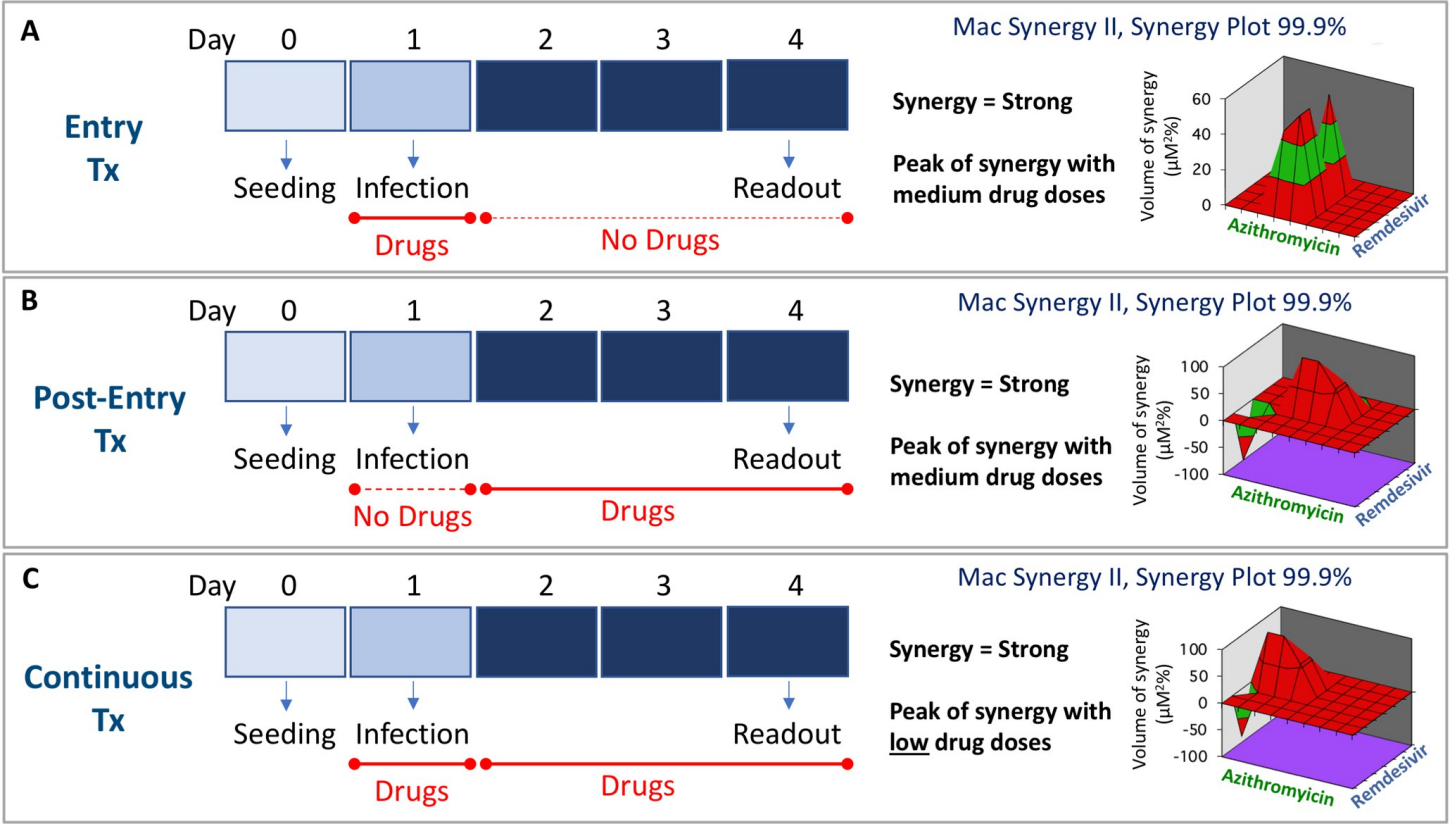
Mechanism of synergy between RDV and AZI. RDV and AZI synergize both at entry and post-entry level, synergy being optimal with continuous treatment. (A) Drugs were added only at the time of infection; (B) Drugs were added only after infection; (C) Drugs were added throughout the experiment. In all cases Vero E6 cells were seeded at Day 0, infection was performed at Day 1 and readout was measured at Day 4. The results of the Mac Synergy II, Synergy Plot 99.9% analyses are shown in the right part of each panel. Tx = treatment.

As RDV, AZI and IVM belong to three different classes of drugs, we asked why these drugs, and not others, would reciprocally synergize among themselves. We postulated the inhibition of common SARS-CoV-2 replication pathways and looked for evidence in the literature. We found that RDV, AZI and IVM might interfere with SARS-CoV-2 by two mechanisms of action (MoA) that are common to all three drugs. One MoA would be at the virus entry/binding level: in fact, RDV has been shown to interact with S protein, ACE2 and TMPRSS2 [26, 27], AZI with S protein, ACE2 and GM1 [28, 29], and IVM with S protein and ACE2 [26]. The other common MoA would be at the RNA replication level: RDV is a nucleoside analog and a well-known inhibitor of the RNA-dependent RNA polymerase (RdRp) [30]; AZI and IVM are both macrolides that have been shown to inhibit RdRp [31] and AZI (and by extension, macrolides) could carry Zn to inhibit RdRp [32]. Next, we performed a time-course experiment by adding RDV and AZI only during infection (entry treatment), only after infection (post-entry treatment) or throughout the entire experiment (continuous treatment), as illustrated in **Fig 5**. We found strong synergy in each of the conditions tested, however, the continuous treatment resulted in synergy at lower drug concentration compared to the other two conditions. Our results are consistent with the entry/binding and post-entry (RdRp) mechanisms of synergy proposed in the literature and indicate that optimal synergy is obtained by taking advantage of both mechanisms.

To completely suppress viral replication and address the unmet need of hospitalized patients we developed broad-spectrum-antivirals-based cocktails for the treatment of patients with COVID-19. We showed that RDV combined with either AZI or IVM leads to a significant synergy that substantially lowers the concentration of each of the three drugs that is required for complete SARS-CoV-2 suppression, thus overcoming the problem of suboptimal drug exposure. Achieving viral suppression with either RDV, AZI, or IVM as monotherapy would in fact require drug concentrations that are too high to be attainable in humans, however, combining RDV with either AZI or IVM lowers the LIC_100_ (that is the lowest drug concentration required for complete viral suppression) to levels that are attainable in the human plasma and/or in the lung. In the case of the lung, which is the most relevant target for a respiratory virus like SARS-CoV-2, such levels are likely to be maintained for one or more days after a single administration of RDV combined with either AZI or IVM.

Other approaches have been explored to overcome the problem of suboptimal drug exposure. If a drug has a high LIC_100_ and such concentration cannot be reached in the human plasma or in the organs by using dosages with acceptable toxicity profiles (or can be reached but can be maintained only for a short time), one can adopt different measures such as: a) to increase drug dosage, at risk of toxicity; b) to chemically modify the drug to prolong the time of release (extended-release) or to decrease its catabolism (in both cases a lengthy and expensive procedure that requires a new regulatory pathway), c) to add another boosting drug in order increase its pharmacological exposure by interfering with its metabolism (e.g. slowing down lopinavir metabolism by adding ritonavir as both drugs compete for cytochrome P4503A4). A similar approach has been recently followed by combining ritonavir with PF-07321332, a new protease inhibitor reported being effective to inhibit SARS-CoV-2, decrease viral load and prevent COVID-19 progression [33].

We followed an alternative approach of lowering a drug’s LIC_100_ by combining it with another broad-spectrum antiviral that bears a synergistic activity. This strategy presents several advantages: a) lowering the LIC_100_ means that the drug will be active at a lower concentration that can be reached in the human plasma or in the lung by using indicated dosages and b) inhibition of virus replication can be maintained for a longer time, without increased toxicity of the drugs, as long as they do not have synergistic cytotoxicity (as it is the case with the combinations described here) and clinicians (who are familiar with these drugs) do not foresee overlapping side effects, c) no requirement of chemical modification or alteration of PK/PD profiles is required, d) no need for dose (and toxicity) increase, on the contrary flexibility to reduce the dose (and toxicity).

We recognize that the broad-spectrum antivirals that we have tested here have already been proposed as anti-SARS-CoV-2 drugs and have shown inconsistent results [34]. We believe there are at least two explanations for such inconsistency: 1) most of the time they have been used alone and 2) when used in combination, such combinations have not been screened rigorously. Our strategy to select highly active antiviral cocktails differs from previous attempts to *in vitro* identify anti-SARS-CoV-2 drugs that have subsequently failed in the clinic [34] for the fact that we adopted highly stringent criteria to test drug combinations: a) we screened for synergy with the rigorous and well-established model MacSynergy II, b) we focused on achieving the lowest drug concentration inhibiting 100% of SARS-CoV-2 (LIC_100_) as opposed to the less stringent IC_50_ or IC_90_ parameters, c) we determined whether the LIC_100_ could be reached in the plasma and in the lung at clinically indicated doses of the drugs, a key analysis that has not been attempted before and d) we specifically focused only on combinations with no observed increase in cytotoxicity, leading to significant improvements in their respective Therapeutic Indices. Moreover, based on their respective package inserts we are not aware of serious overlapping potential toxicity between RDV and either AZI or IVM that would preclude their combinations *in vivo* and we have not found any references in the literature that would challenge this assumption. These three drugs have established toxicity profiles and have been extensively used to treat patients with infectious diseases. For example, antibiotics are often administered to hospitalized COVID-19 patients and AZI could become the 1^st^ line antibiotic in this setting. Our results would suggest the possibility of once-a-day (QD) administration may be effective for any of these combinations. A clinical study submitted after our study had been submitted and published while our manuscript was under review found that the coadministration of RDV and AZI was associated with reduced risk of ICU admission, independently of covariates [17].

In short, our results 1) explain why add-on of repurposed drugs have mostly failed to induce beneficial clinical effects during SARS-CoV-2 infection when used as monotherapies; 2) suggest that some of these drugs could be selected to generate effective drug combinations; 3) provide a validated model for testing drugs *in vitro* looking for synergy and calculating active drug levels that are achievable *in vivo*, thus predicting which drug combinations might be most effective in the clinics; 4) elucidate the MoA of these combinations.

Combining drugs against SARS-CoV-2 would be in line with the treatment of several other viral infections, such as the Human Immunodeficiency Virus (HIV) and Hepatitis C infection, also to prevent the development of resistant mutants [14]. All proposed RDV-based cocktails described here contain broad-spectrum antivirals and their improved antiviral activity does not appear to be strain-specific, therefore each of these drug combinations could be therapeutically active in the plasma and the lung against present and emerging coronavirus variants and perhaps against a broad range of other viruses such as RSV, Marburg virus, Ebola virus and West Nile virus. There is sufficient evidence to support performing randomized, controlled, prospective clinical trials for conclusive confirmation of the efficacy and toxicity of the proposed cocktails.
