# Correction: Synergistic drug combinations designed to fully suppress SARS-CoV-2 in the lung of COVID-19 patients

**DOI:** 10.1371/journal.pone.0315297

**Published:** 2024-12-05

**Authors:** Davide De Forni, Barbara Poddesu, Giulia Cugia, Daniela Pagnozzi, James Chafouleas, Julianna Lisziewicz, Franco Lori

Daniela Pagnozzi should be included in the author byline. Daniela Pagnozzi should be listed as the fourth author, and affiliated with 2: Porto Conte Ricerche s.r.l., Tramariglio, Italy. The contributions of this author are as follows: managed and maintained research data, secured financial support, developed the methodology, and created the review and performed the revision.

The correct citation is: De Forni D, Poddesu B, Cugia G, Pagnozzi D, Chafouleas J, Lisziewicz J (2022) Synergistic drug combinations designed to fully suppress SARS-CoV-2 in the lung of COVID-19 patients. PLOS ONE 17(11): e0276751. https://doi.org/10.1371/journal.pone.0276751

The following information is missing from the Funding statement: This work has also been funded by Sardegna Ricerche (art. 9 LR 20/2015).

There are errors in the Acknowledgement statement. The correct Acknowledgement statement is as follows: We thank Sjlva Petrocchi for editing the manuscript. We thank the Department of Molecular and Translational Medicine, Section of Microbiology and Virology, University of Brescia Medical School in Brescia, Italy and the Istituto Lazzaro Spallanzani in Rome, Italy for providing the SARS-CoV-2 strains.

## References

[1] De Forni D, Poddesu B, Cugia G, Chafouleas J, Lisziewicz J, Lori F (2022) Synergistic drug combinations designed to fully suppress SARS-CoV-2 in the lung of COVID-19 patients. PLOS ONE 17(11): e0276751. doi: 10.1371/journal.pone.0276751 36355808 PMC9648746

